# A New Random Forest Algorithm-Based Prediction Model of Post-operative Mortality in Geriatric Patients With Hip Fractures

**DOI:** 10.3389/fmed.2022.829977

**Published:** 2022-05-11

**Authors:** Fei Xing, Rong Luo, Ming Liu, Zongke Zhou, Zhou Xiang, Xin Duan

**Affiliations:** Department of Orthopedics, Orthopedic Research Institute, West China Hospital, Sichuan University, Chengdu, China

**Keywords:** machine learning, random forest, hip fracture, mortality, prediction model

## Abstract

**Background:**

Post-operative mortality risk assessment for geriatric patients with hip fractures (HF) is a challenge for clinicians. Early identification of geriatric HF patients with a high risk of post-operative death is helpful for early intervention and improving clinical prognosis. However, a single significant risk factor of post-operative death cannot accurately predict the prognosis of geriatric HF patients. Therefore, our study aims to utilize a machine learning approach, random forest algorithm, to fabricate a prediction model for post-operative death of geriatric HF patients.

**Methods:**

This retrospective study enrolled consecutive geriatric HF patients who underwent treatment for surgery. The study cohort was divided into training and testing datasets at a 70:30 ratio. The random forest algorithm selected or excluded variables according to the feature importance. Least absolute shrinkage and selection operator (Lasso) was utilized to compare feature selection results of random forest. The confirmed variables were used to create a simplified model instead of a full model with all variables. The prediction model was then verified in the training dataset and testing dataset. Additionally, a prediction model constructed by logistic regression was used as a control to evaluate the efficiency of the new prediction model.

**Results:**

Feature selection by random forest algorithm and Lasso regression demonstrated that seven variables, including age, time from injury to surgery, chronic obstructive pulmonary disease (COPD), albumin, hemoglobin, history of malignancy, and perioperative blood transfusion, could be used to predict the 1-year post-operative mortality. The area under the curve (AUC) of the random forest algorithm-based prediction model in training and testing datasets were 1.000, and 0.813, respectively. While the prediction tool constructed by logistic regression in training and testing datasets were 0.895, and 0.797, respectively.

**Conclusions:**

Compared with logistic regression, the random forest algorithm-based prediction model exhibits better predictive ability for geriatric HF patients with a high risk of death within post-operative 1 year.

## Introduction

The prevalence of geriatric hip fracture (HF) patients is increasing in the rapidly aging population, which has become a growing public health concern worldwide (1–4). In addition, geriatric HF patients are associated with high post-operative mortality (5, 6). Previous studies revealed that the overall post-operative mortality of geriatric HF patients in 1 year was as high as 31% (7, 8). The construction of a reliable post-operative mortality prediction model based on risk factors can be applied in the early identification of geriatric HF patients with a high risk of post-operative death, which plays a vital role in taking timely interventions to avoid post-operative death (9, 10). However, the previous prediction model constructed by logistic regression exhibits low accuracy in identifying geriatric HF patients with a high risk of post-operative death.

As a subset of machine learning algorithms, random forest algorithm can build a mathematical model based on sample data and be used to make predictions or decisions (11–13). The previous studies demonstrated that the prediction model based on random forest algorithm exhibited a high accuracy in predicting the development of end-stage renal disease (14). Additionally, the prediction model based on random forest algorithm can provide essential insights to clinical doctors who can then adapt their diagnosis and treatment for patients by predicting risks in advance. Accordingly, we constructed a machine learning model to predict 1-year post-operative mortality of geriatric HF patients by identifying the risk factors. In addition, we evaluated the efficiency of the random forest algorithm-based prediction model by comparing it with traditional logistic regression.

## Methods

### Study Design and Data Collection

This retrospective observational cohort study was based on data collected from January 2013 to August 2017 in West China Hospital. The inclusion criteria for this study were as follows: (1) HF patients older than 60 years. (2) Patients underwent surgical treatments. The exclusion criteria for this study were as follows: (1) High energy trauma mechanism. (2) Secondary fracture. (3) Multiple fractures. (4) Open fractures. (5) Old fracture (>7 days). (6) Pathological fracture. (7) Abdominal organ injury. (8) Incomplete clinical data. A total of 591 geriatric HF patients were enrolled in this study. Then, all cases were randomly divided into training and testing datasets at a ratio of 70:30. The patients were diagnosed with HF using physical examination combined with medical imaging (X-rays or computed tomography). The case data, including demographic variables (gender, age, BMI, injury side, type of fracture, time from injury to surgery, type of surgery, operation duration, perioperative blood transfusion, blood loss during surgery, hospital stays), medical history (smoking, history of malignancy, history of cerebrovascular disease), comorbidities (chronic obstructive pulmonary disease, diabetes, hypertension, renal dysfunction, liver disease, HIV/AIDS), laboratory tests (hemoglobin, blood platelet, leukocyte, albumin, serum potassium, serum sodium), and 1-year post-operative mortality, were extracted by several clinicians who had received a standardized training. The correlation analysis of all variables was also conducted.

### Random Forest Modeling

**Figure 1:**
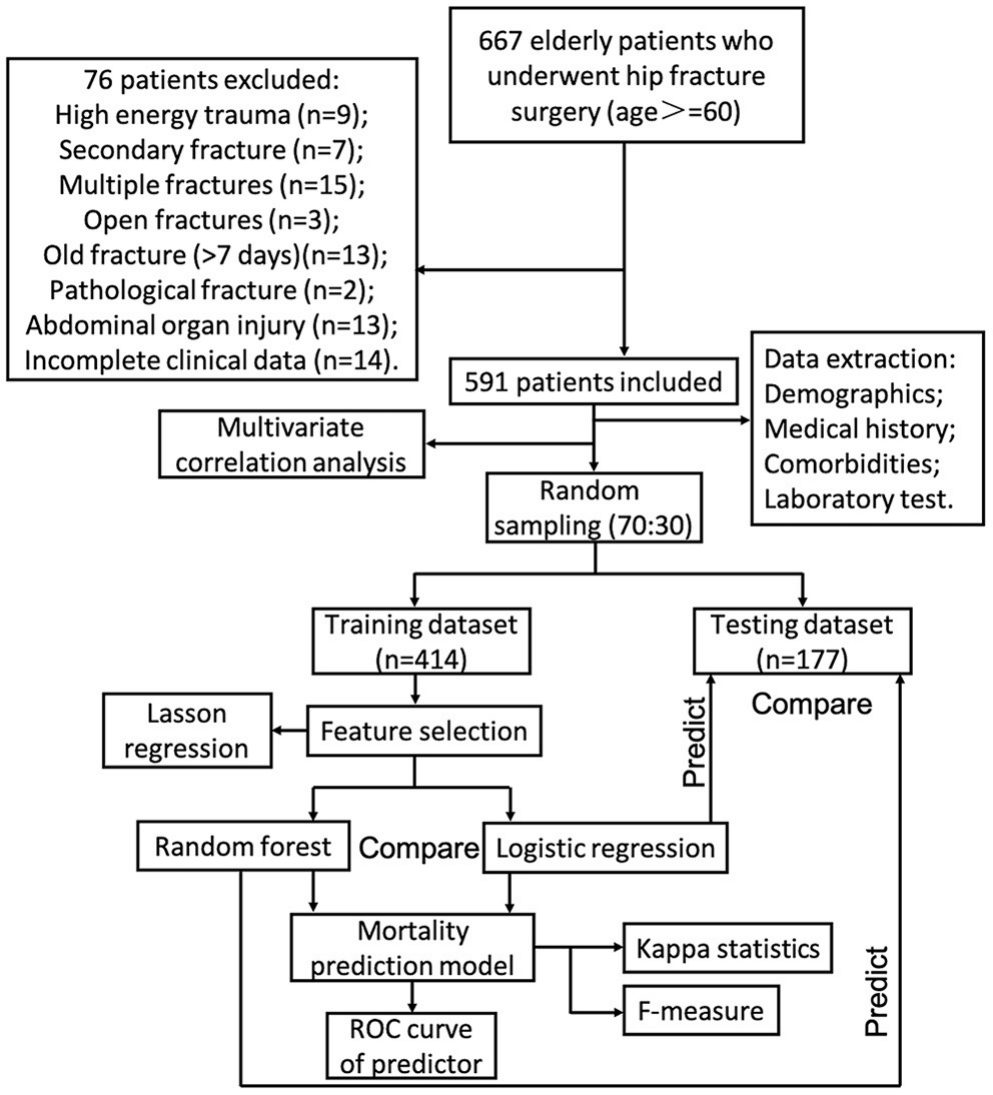
The procedure of establishing mortality prediction models in this study.

Age, BMI, time from injury to surgery, operation duration, blood loss during surgery, hospital stays, and laboratory tests were entered into the random forest procedure as continuous variables. Gender, injury side, type of fracture, type of surgery, injury side, type of fracture, type of surgery, perioperative blood transfusion, medical history, and comorbidities were entered as dichotomous variables. The data were sampled by using the random bootstrapping/bagging method. According to the characteristics of the numerous variables, classification and regression trees were performed to the classified training dataset. The input variables in the risk prediction model were ranked based on the mean decrease in accuracy and the mean decrease in the Gini coefficient. Approximately one-third of the entire data set was not randomly sampled; this out-of-bag (OOB) data served as the testing set. The number of decision trees was set at 500. The random forest algorithm was performed to select variables and create a risk prediction model. Lasso binary logistic regression was performed to compare feature selection and regularization results of the random forest algorithm. The risk prediction model constructed by random forest algorithm was then verified in the training dataset and testing dataset, respectively. Logistic regression is a linear fit of a response variable to a logarithmic probability ratio (15, 16). The aim of classification by logistic regression is to establish a regression formula to classify boundary lines based on existing data. As a control, the risk prediction model constructed by logistic regression was also verified in the training dataset and testing dataset. Finally, the area under curve (AUC) of continuous variables, random forest algorithm, and traditional logistic regression were also calculated. In addition, the Kappa statistic and *F*-measure were also used to test model reliability. The procedure of establishing mortality prediction models in this study is shown in Figure 1.

### Statistical Analysis

Descriptive statistics of continuous variables were expressed as mean, and the categorical variables were reported as numbers and percentages. Independent *t*-test was performed to compare continuous variables and chi-square test for categorical variables. All the statistical analyses in this study were performed using the RStudio (version 0.99, Boston, United States) statistical software package, which runs R software (version 3.5.1). RStudio with library packages *Boruta* (CRAN.R-project.org/package=Boruta), *randomforest* (CRAN.R-project.org/package=randomForest), and *caret* (CRAN.R-project.org/package=caret) were used to construct a random forest model. In addition, RStudio with library packages *glmnet* (CRAN.R-project.org/package=glmnet) was used for Lasso regression. *P*-values < 0.05 were considered statistically significant.

## Results

### Patient Characteristics

**Figure 2:**
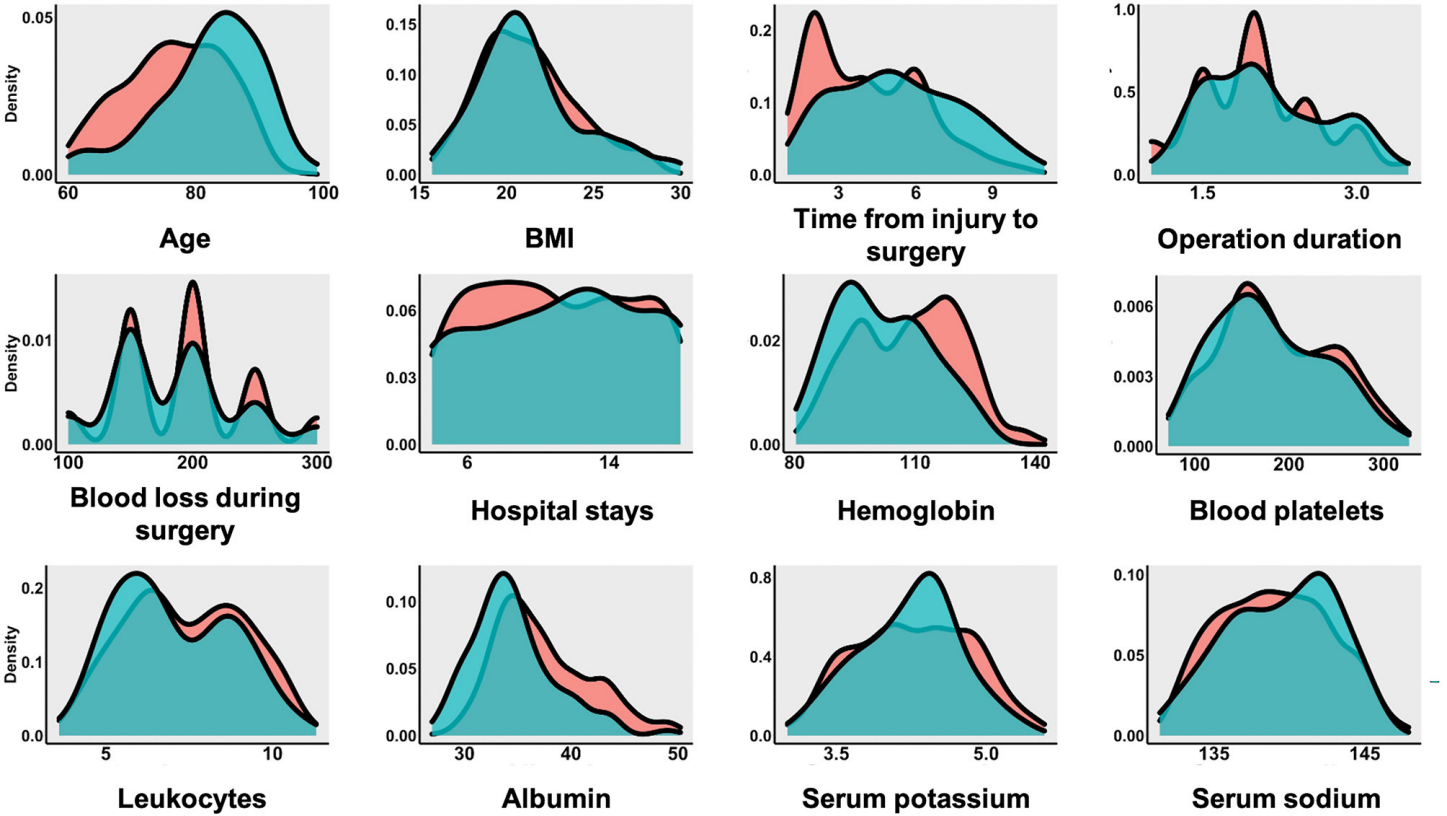
The continuous variables distribution of live group and dead group.

The baseline characteristics of all enrolled patients are shown in Table 1. The 1-year post-operative mortality rate of all enrolled HF patients was 14.72%. The continuous variables distribution of the live and dead groups is shown in Figure 2. The mean age of all enrolled HF patients was 77.40 ± 8.25 years. The age distribution differed significantly between the live and dead groups (*P* < 0.001). The time from injury to surgery of all enrolled patients was 4.29 ± 2.26 days. Additionally, the time from injury to surgery in the live group was significantly lower than that of the dead group. The laboratory results revealed that the hemoglobin and albumin of the dead group were significantly lower than those of the live group.

**Table 1:** The baseline characteristics of all enrolled patients.

**Variables**	**Total patients** **(*n* = 591)**	**Live group** **(*n* = 504)**	**Dead group** **(*n* = 87)**	***P*** **-value**
**Demographics**				
Gender				0.483
Male	238	200	38	
Female	353	304	49	
Age, years	77.40 ± 8.25	76.58 ± 7.98	82.15 ± 8.21	<0.001
BMI, kg/m^2^	21.27 ± 2.85	21.27 ± 2.81	21.30 ± 3.07	0.913
Injury side				0.764
Left	328	281	47	
Right	263	223	40	
Type of fracture				0.730
Intra-articular fracture	316	268	48	
Extra-articular fracture	275	236	39	
Time from injury to surgery, days	4.29 ± 2.26	4.10 ± 2.18	5.36 ± 2.41	<0.001
Surgery type				0.210
Internal fixation	375	325	50	
Joint arthroplasty	216	179	37	
Operation duration, hours	2.05 ± 0.59	2.04 ± 0.59	2.12 ± 0.60	0.206
Perioperative blood transfusion	29	17	12	<0.001
Blood loss during surgery	190.78 ± 50.21	191.87 ± 50.08	184.48 ± 50.76	0.206
Hospital stays	11.06 ± 4.33	11.00 ± 4.27	11.39 ± 4.69	0.445
**Medical history**				
Smoking (current and past)	167	136	31	0.098
History of malignancy	22	15	7	0.045
History of cerebrovascular disease	34	23	11	0.003
**Comorbidities**				
Chronic obstructive pulmonary disease (COPD)	142	94	48	<0.001
Diabetes	127	112	15	0.296
Hypertension	217	190	27	0.234
Renal dysfunction	12	9	3	0.546
Liver disease	16	13	3	0.918
HIV/AIDS	5	5	0	0.206
**Laboratory test**				
Hemoglobin, g/L	107.48 ± 12.94	108.44 ± 12.90	101.87 ± 11.70	<0.001
Blood platelets, 10^9^/L	186.18 ± 60.04	187.33 ± 60.31	179.48 ± 58.33	0.260
Leukocytes, 10^9^/L	7.35 ± 1.77	7.40 ± 1.78	7.05 ± 1.73	0.091
Albumin, g/L	36.99 ± 4.54	37.40 ± 4.50	34.60 ± 3.98	<0.001
Serum potassium, mmol/L	4.27 ± 0.57	4.28 ± 0.58	4.24 ± 0.50	0.482
Serum sodium, mmol/L	139.10 ± 3.64	139.04 ± 3.66	139.43 ± 3.55	0.367

**Figure 3:**
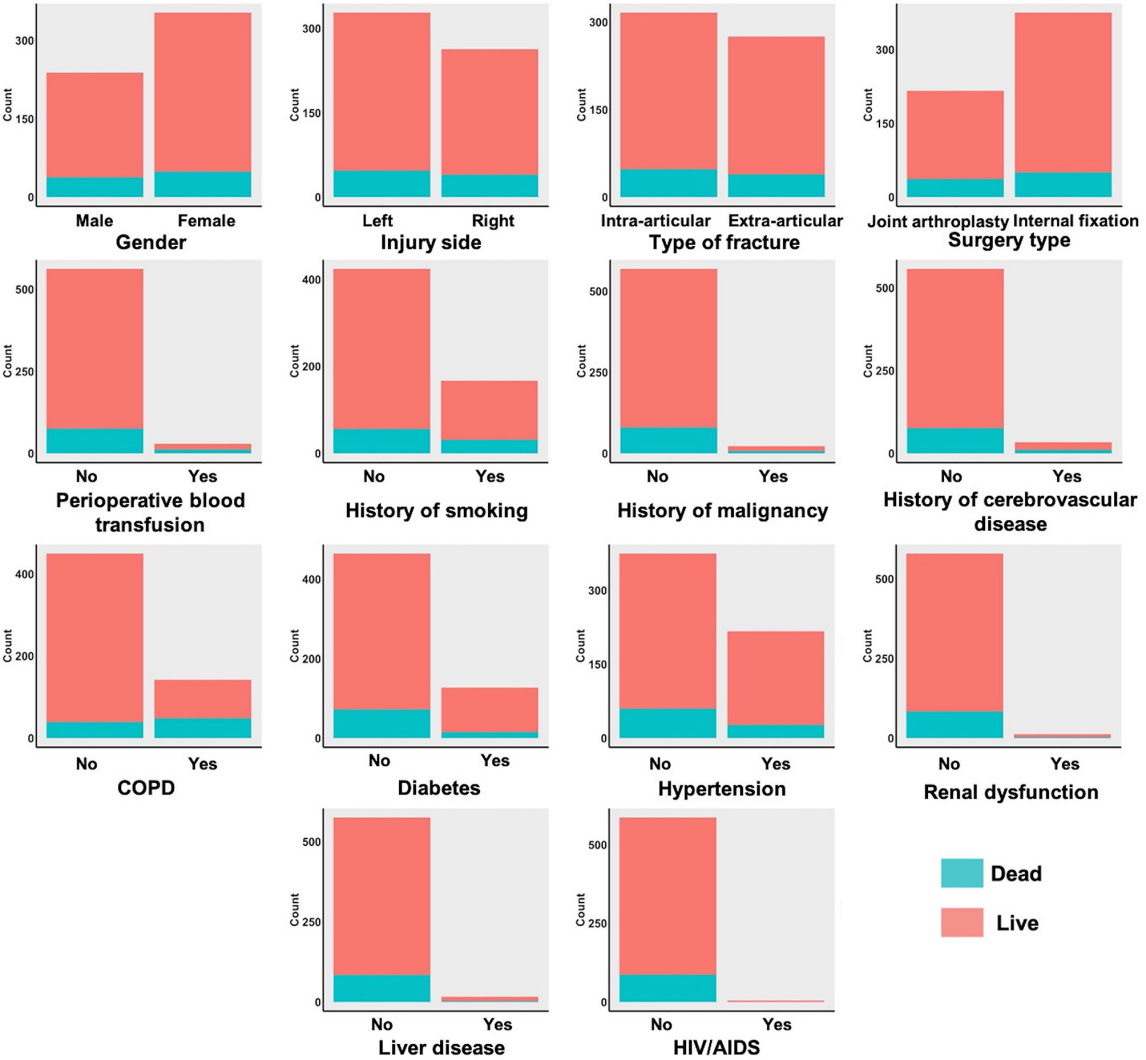
The dichotomous variables of live group and dead group.

**Figure 4:**
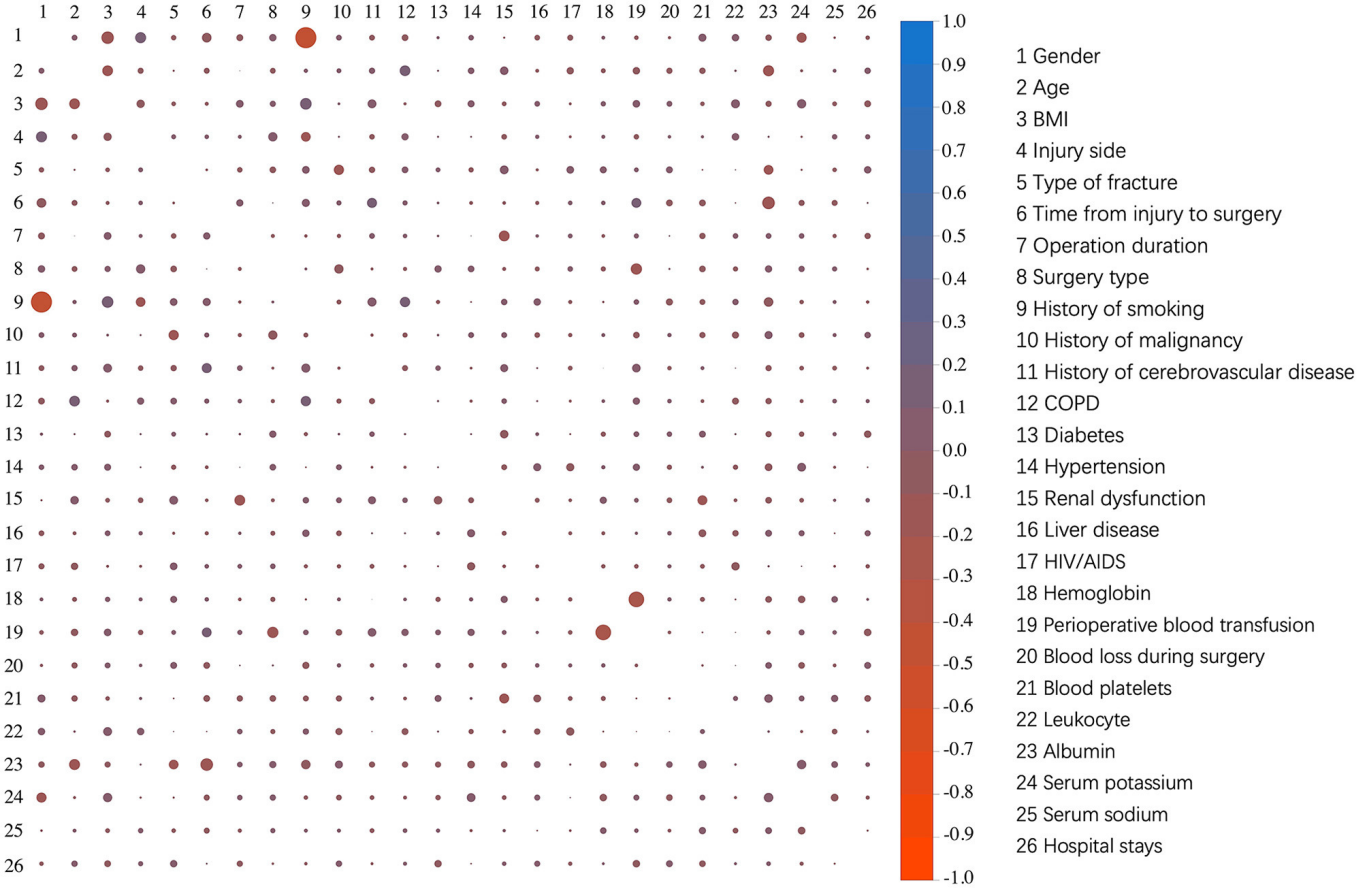
The correlation analysis results of all variables.

The dichotomous variables of the live and dead groups are shown in Figure 3. Among these patients, 316 patients were diagnosed with intra-articular fracture, and 275 patients were diagnosed with extra-articular fracture. Of all the enrolled patients, 167 patients had a history of smoking, 22 patients with a history of malignancy, 34 patients with a history of cerebrovascular disease. Comorbidities in all enrolled patients included 142 patients with chronic obstructive pulmonary disease (COPD), 127 patients with diabetes, 217 patients with hypertension, 12 patients with renal dysfunction, 16 patients with liver disease, and five patients with HIV/AIDS. The presence of COPD was also associated with a higher 1-year post-operative mortality rate. The correlation analysis results of all variables are shown in Figure 4. All cases were randomly divided into training and testing datasets at a ratio of 70:30 (Table 2). The baseline characteristics of training datasets (Table 3) and testing datasets (Table 4) were comparable, consistent with the overall population.

**Table 2:** The baseline characteristics of training dataset and texting dataset.

**Variables**	**Training dataset** **(*n* = 414)**	**Texting dataset** **(*n* = 177)**	***P*** **-value**
**Demographics**				
Gender			0.815
Male	168	70	
Female	246	107	
Age, years	77.09 ± 8.36	78.12 ± 7.97	0.166
BMI, kg/m^2^	21.26 ± 2.86	21.30 ± 2.83	0.853
Injury side			0.686
Left	232	96	
Right	182	81	
Type of fracture			0.635
Intra-articular fracture	224	92	
Extra-articular fracture	190	85	
Time from injury to surgery, days	4.22 ± 2.26	4.45 ± 2.27	0.260
Surgery type			0.616
Internal fixation	260	115	
Joint arthroplasty	154	62	
Operation duration, hours	2.05 ± 0.58	2.07 ± 0.61	0.680
Perioperative blood transfusion	23	6	0.264
Blood loss during surgery	190.58 ± 49.04	191.24 ± 52.98	0.883
Hospital stays	11.20 ± 4.37	10.73 ± 4.24	0.229
**Medical history**				
Smoking (current and past)	123	41	0.230
History of malignancy	12	10	0.106
History of cerebrovascular disease	24	10	0.944
**Comorbidities**				
Chronic obstructive pulmonary disease (COPD)	93	49	0.174
Diabetes	94	33	0.271
Hypertension	142	75	0.062
Renal dysfunction	11	1	0.182
Liver disease	12	4	0.872
HIV/AIDS	4	1	0.612
**Laboratory test**				
Hemoglobin, g/L	107.15 ± 12.78	108.23 ± 13.30	0.353
Blood platelets, 10^9^/L	189.64 ± 60.68	178.07 ± 57.87	0.032
Leukocytes, 10^9^/L	7.52 ± 1.74	6.93 ± 1.79	0.001
Albumin, g/L	36.83 ± 4.42	37.33 ± 4.78	0.228
Serum potassium, mmol/L	4.28 ± 0.56	4.24 ± 0.58	0.392
Serum sodium, mmol/L	139.02 ± 3.67	139.28 ± 3.58	0.443

**Table 3:** The baseline characteristics of the training dataset.

**Variables**	**Total patients** **(*n* = 414)**	**Live group** **(*n* = 353)**	**Dead group** **(*n* = 61)**	***P*** **-value**
**Demographics**				
Gender				0.526
Male	168	141	27	
Female	246	212	34	
Age, years	77.09 ± 8.36	76.24 ± 8.09	82.00 ± 8.28	<0.001
BMI, kg/m^2^	21.26 ± 2.86	21.24 ± 2.86	21.33 ± 2.91	0.828
Injury side				0.741
Left	232	199	33	
Right	182	154	28	
Type of fracture				0.266
Intra-articular fracture	224	187	37	
Extra-articular fracture	190	166	24	
Time from injury to surgery, days	4.22 ± 2.26	4.04 ± 2.17	5.26 ± 2.46	<0.001
Surgery type				0.036
Internal fixation	260	229	31	
Joint arthroplasty	154	124	30	
Operation duration, hours	2.05 ± 0.58	2.03 ± 0.58	2.15 ± 0.57	0.139
Perioperative blood transfusion	23	14	9	0.002
Blood loss during surgery	190.58 ± 49.04	192.21 ± 48.88	181.15 ± 49.30	0.104
Hospital stays	11.20 ± 4.37	11.12 ± 4.31	11.67 ± 4.66	0.364
**Medical history**				
Smoking (current and past)	123	99	24	0.075
History of malignancy	12	6	6	0.002
History of cerebrovascular disease	24	18	6	0.244
**Comorbidities**				
Chronic obstructive pulmonary disease (COPD)	93	59	34	<0.001
Diabetes	94	84	10	0.203
Hypertension	142	124	18	0.393
Renal dysfunction	11	8	3	0.448
Liver disease	12	9	3	0.545
HIV/AIDS	4	4	0	0.258
**Laboratory test**				
Hemoglobin, g/L	107.15 ± 12.78	108.18 ± 12.78	101.20 ± 11.16	<0.001
Blood platelets, 10^9^/L	189.64 ± 60.68	190.82 ± 60.17	182.82 ± 63.62	0.342
Leukocytes, 10^9^/L	7.52 ± 1.74	7.55 ± 1.74	7.36 ± 1.70	0.414
Albumin, g/L	36.83 ± 4.42	37.22 ± 4.38	34.64 ± 4.08	<0.001
Serum potassium, mmol/L	4.28 ± 0.56	4.29 ± 0.57	4.26 ± 0.50	0.704
Serum sodium, mmol/L	139.02 ± 3.67	138.96 ± 3.67	139.42 ± 3.64	0.365

**Table 4:** The baseline characteristics of the texting dataset.

**Variables**	**Total patients** **(*n* = 177)**	**Live group** **(*n* = 151)**	**Dead group** **(*n* = 26)**	***P*** **-value**
**Demographics**				
Gender				0.755
Male	70	59	11	
Female	107	92	15	
Age, years	78.12 ± 7.97	77.36 ± 7.70	82.50 ± 8.20	0.002
BMI, kg/m^2^	21.30 ± 2.83	21.32 ± 2.72	21.23 ± 3.47	0.888
Injury side				0.965
Left	96	82	14	
Right	81	69	12	
Type of fracture				0.285
Intra-articular fracture	92	81	11	
Extra-articular fracture	85	70	15	
Time from injury to surgery, days	4.45 ± 2.27	4.25 ± 2.21	5.58 ± 2.30	0.006
Surgery type				0.348
Internal fixation	115	96	19	
Joint arthroplasty	62	55	7	
Operation duration, hours	2.07 ± 0.61	2.07 ± 0.60	2.07 ± 0.67	0.935
Perioperative blood transfusion	6	3	3	0.058
Blood loss during surgery	191.24 ± 52.98	191.06 ± 52.94	192.30 ± 54.21	0.912
Hospital stays	10.73 ± 4.24	10.73 ± 4.16	10.73 ± 4.77	0.996
**Medical history**				
Smoking (current and past)	41	34	7	0.792
History of malignancy	10	9	1	0.999
History of cerebrovascular disease	10	5	5	0.005
**Comorbidities**				
Chronic obstructive pulmonary disease (COPD)	49	35	14	0.001
Diabetes	33	28	5	0.934
Hypertension	75	66	9	0.386
Renal dysfunction	1	1	0	0.572
Liver disease	4	4	0	0.257
HIV/AIDS	1	1	0	0.572
**Laboratory test**				
Hemoglobin, g/L	108.23 ± 13.30	109.06 ± 13.22	103.42 ± 12.97	0.049
Blood platelets, 10^9^/L	178.07 ± 57.87	179.18 ± 60.04	171.65 ± 43.59	0.542
Leukocytes, 10^9^/L	6.93 ± 1.79	7.04 ± 1.81	6.33 ± 1.59	0.062
Albumin, g/L	37.33 ± 4.78	37.82 ± 4.77	34.52 ± 3.81	<0.001
Serum potassium, mmol/L	4.24 ± 0.58	4.25 ± 0.59	4.17 ± 0.51	0.533
Serum sodium, mmol/L	139.28 ± 3.58	139.25 ± 3.62	139.44 ± 3.39	0.796

### Feature Selection

**Figure 5:**
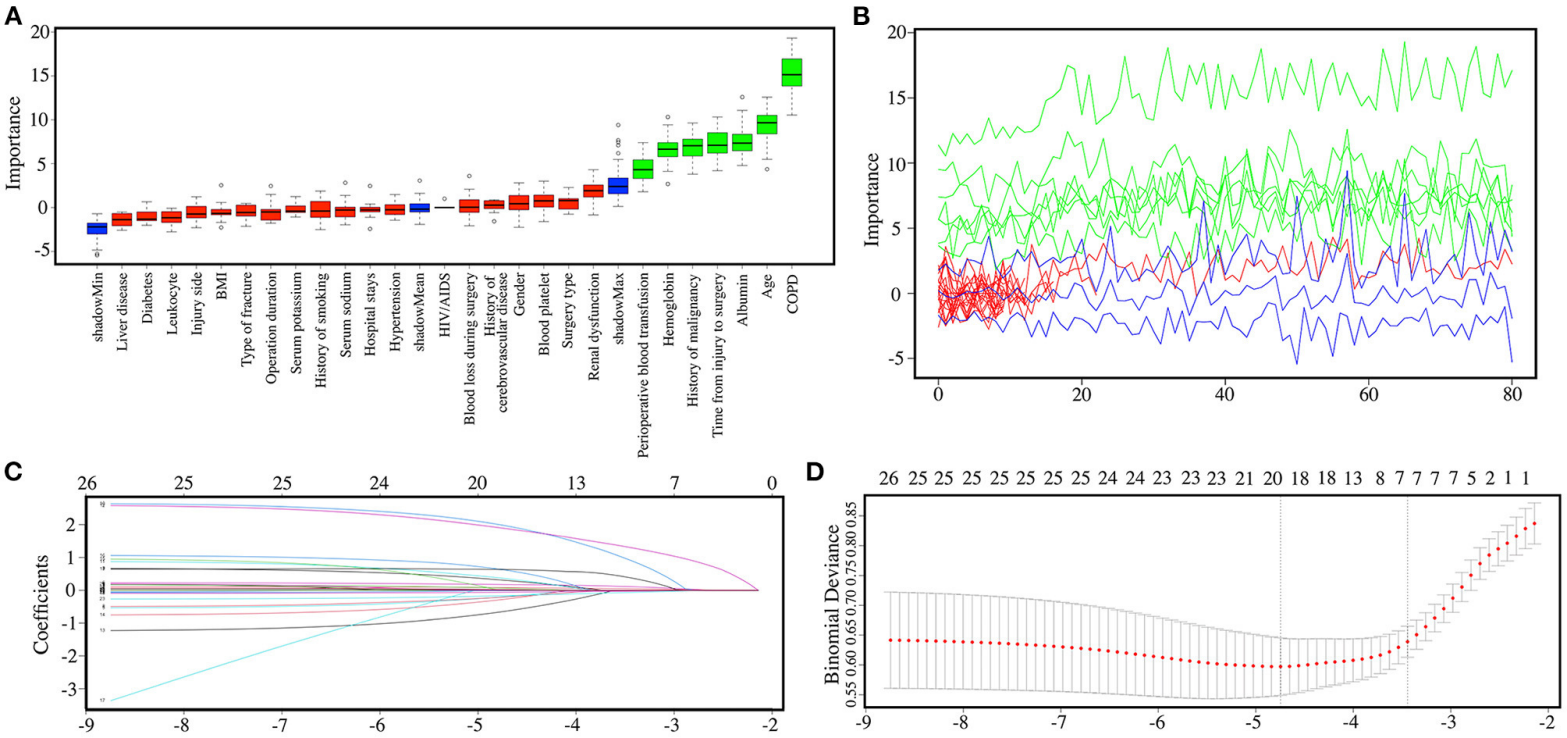
**(A)** The boxplot reveals the importance of each of the individual variables in random forest algorithm. Boxplots in green, yellow, and blue were confirmed as important, tentative, and unimportant variables, respectively. **(B)** Decisions of rejecting or accepting features by random forest in 100 Boruta function runs. **(C)** Lasso coefficient profiles of all features. **(D)** The tuning parameter λ (lambda) selection in the Lasso regression model used 10-fold cross-validation by minimum criteria.

Using the random forest prediction model, all variables were tested for their ability to predict the post-operative 1-year death in geriatric HF patients. Figures 5A,B show the procedure and results of feature selection by random forest algorithm. The feature selection results by random forest algorithm revealed that five variables, including age, time from injury to surgery, COPD, albumin, hemoglobin, history of malignancy, and perioperative blood transfusion, could be used to predict the 1-year post-operative mortality. Additionally, the Lasso regression model was performed in this study to identify feature selection and regularization results of the random forest algorithm. The Lasso coefficient profiles of features and the optimal penalization coefficient lambda (λ) are shown in Figures 5C,D. The feature selection results of Lasso regression show five variables were associated with 1-year post-operative mortality in geriatric HF patients, which was consistent with the feature selection results by random forest algorithm.

### Random Forest Algorithm-Based Prediction Model

**Figure 6:**
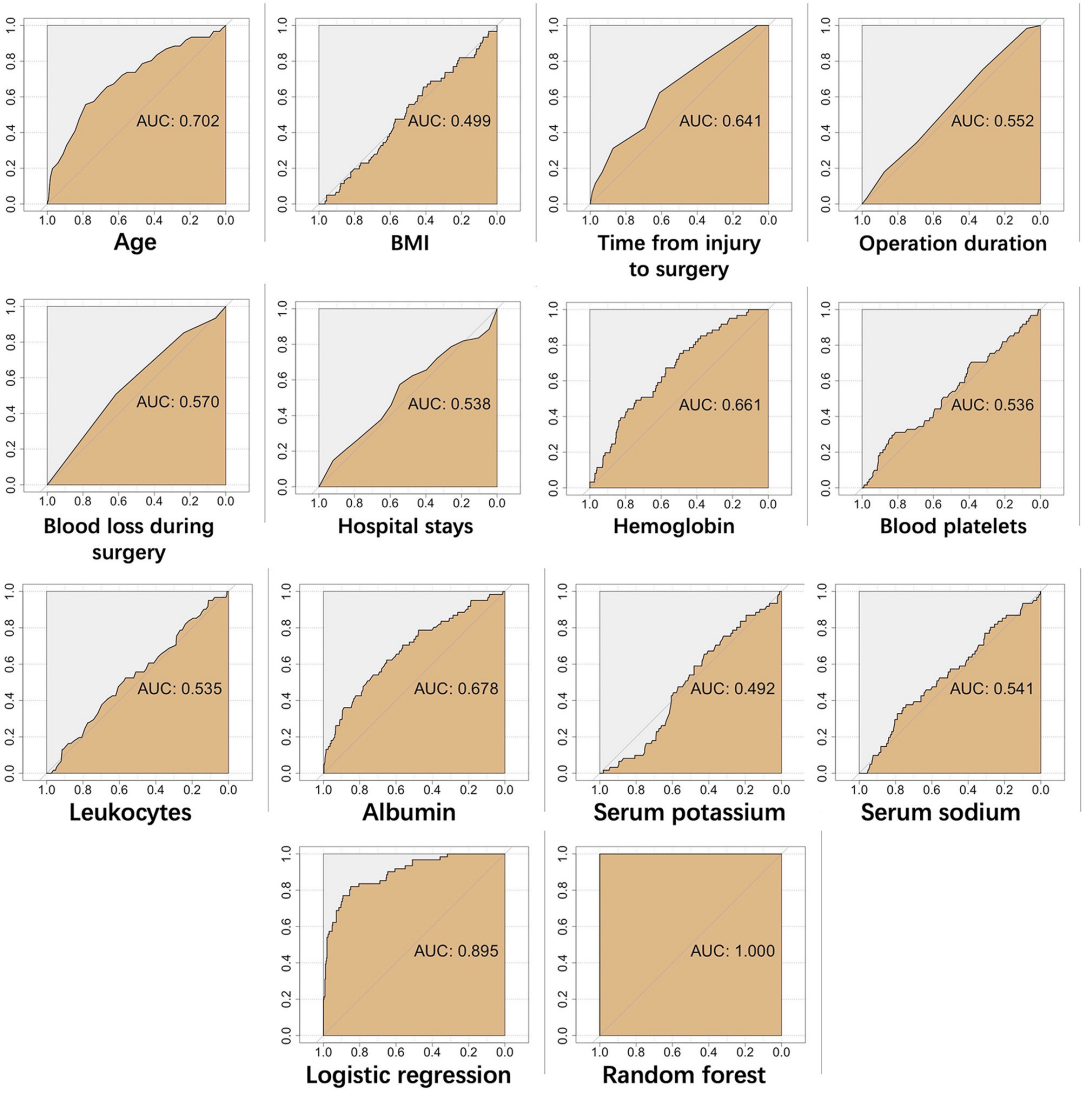
The ROC curves of continuous variables, prediction model constructed by random forest algorithm, and traditional logistic regression in training dataset.

**Figure 7:**
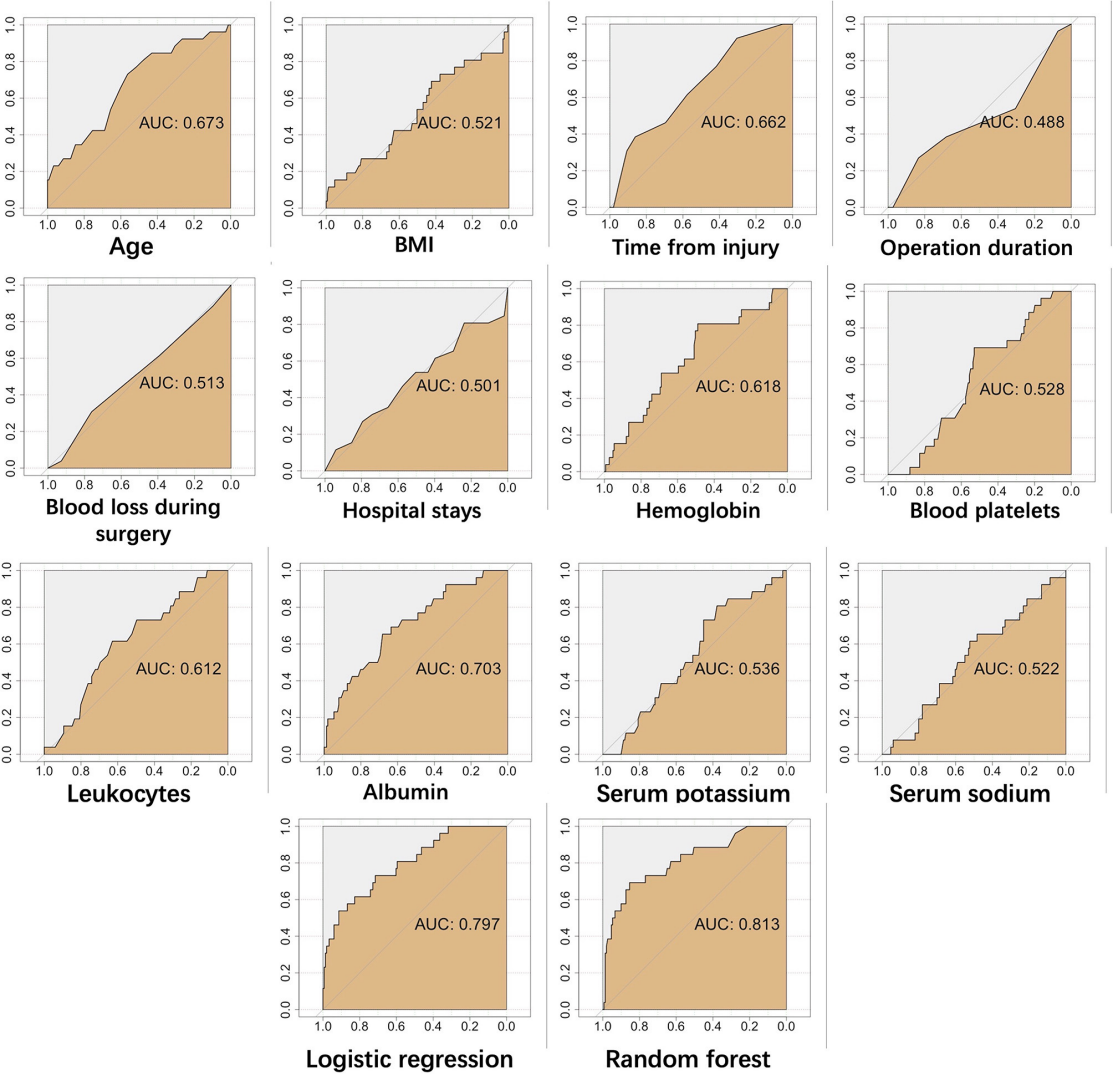
The ROC curves of continuous variables, prediction model constructed by random forest algorithm, and traditional logistic regression in testing dataset.

A risk prediction model was constructed based on confirmed important risk factors selected by the random forest algorithm. In the three runs with mtry values of three, four, or five, we obtained the best result of four, with a low OOB error rate of 14.49%. The ROC curves of continuous variables, prediction model constructed by random forest algorithm, and traditional logistic regression in the training dataset and testing dataset are shown in Figures 6, 7. The area under the curve (AUC) of the random forest algorithm-based prediction model in the training dataset and the testing dataset was 1.000, and 0.813, respectively, which confirmed the good discrimination performance of the prediction model. Additionally, the AUC of the risk prediction model constructed by logistic regression in the training dataset and the testing dataset was 0.895, and 0.797, respectively.

The Kappa statistic and *F*-measure were applied in testing the reliability of prediction models in our study. The Kappa values of random forest algorithm-based prediction model and logistic regression-based prediction model in the training dataset were 1.000 and 0.521, respectively. The Kappa values of random forest algorithm-based prediction model and logistic regression-based prediction model in the testing dataset were 0.488 and 0.267, respectively. The *F*-measures of random forest algorithm-based prediction model and logistic regression-based prediction model in the training dataset were 1.000 and 0.610, respectively. The F-measures of random forest algorithm-based prediction model and logistic regression-based prediction model in the testing dataset were 0.560 and 0.413, respectively.

## Discussion

With the aging population, the number of HF patients is predicted to increase (17). Compared with younger HF patients, geriatric HF patients have more comorbidities and seem to be at higher risk for post-operative death (18). For geriatric HF patients with a higher risk of death after surgery, treatment is not limited to surgery but should also include long-term personalized care at home. Early identification of geriatric HF patients with a high risk of post-operative death is helpful for early intervention and improving clinical prognosis. In the present study, we constructed a reliable risk prediction model with high discriminatory ability, which is helpful in building personalized treatment plans for geriatric HF patients with a high risk of post-operative death.

Most baseline factors of geriatric HF patients between live and dead groups were significantly different, so it is possible to use baseline factors at the onset to predict the clinical prognosis of patients. Currently, several risk prediction tools have been constructed to predict the clinical prognosis of geriatric HF patients (17, 19–21). However, all these prediction tools were constructed by typically performed univariate regression followed by multivariate logistic regression, resulting in reduced prediction accuracy. As a kind of machine learning algorithm, the random forest algorithm, proposed by Breiman in 2001, is an ensemble learning method for classification and regression (22). Random forest algorithm is performed by constructing a multitude of decision trees at training time and outputting the class that is the mode of the classes (classification) or mean prediction (regression) of the individual trees (23). Compared with logistic regression, the random forest algorithm does not require strict assumptions about raw data and has a higher accuracy of disease risk prediction (14, 24). Random forest prediction models can be performed to evaluate the importance of all variables in the procedure of determining categories. Meanwhile, in contrast to traditional prediction models, random forest models have a high ability to handle thousands of input clinical variables and evaluate the missing data to maintain the prediction accuracy (25).

Our study utilized a random forest algorithm to construct a post-operative mortality prediction model based on risk factors. Additionally, the ROC analysis results demonstrated that the random forest algorithm-based prediction model has higher predictive accuracy than logistic regression-based model in training and testing datasets. As far as we know, this study is the first attempt to utilize the random forest to predict the post-operative clinical prognosis of geriatric HF patients. The results of our study demonstrate the potential of a random forest algorithm for predicting the prognosis of geriatric fracture patients. In our opinion, as clinical research based on big data has become a trend, machine learning represented by random forests would be applied to the construction of various disease risk prediction models. The 1-year post-operative mortality is one of the important indicators for evaluating the clinical prognosis of geriatric HF patients (26). Our study reported that the 1-year post-operative mortality of geriatric Chinese HF patients was 14.72%, lower than that of other countries (27, 28). This result may be explained by the fact that the average age and number of comorbidities on admission in our study were lower than those of other countries.

Currently, many previous studies reported that many risk factors could affect post-operative 1-year mortality in geriatric HF patients, such as age, surgery delay, hemoglobin, albumin, serum sodium, C-reactive protein, parathyroid hormone, thyroid-stimulating hormone, renal failure, diabetes, metabolic syndrome (28–40) (Table 5). All these clinical variables were associated with mortality of geriatric HF patients at post-operative different time points. Our study demonstrated that several clinical variables, including age, time from injury to surgery, COPD, albumin, hemoglobin, history of malignancy, and perioperative blood transfusion, were associated with 1-year post-operative mortality in geriatric Chinese HF patients. In our study, age is an independent risk factor of post-operative mortality in geriatric HF patients, consistent with previous studies (18). A possible explanation for this might be that higher age is associated with the poorer preoperative health status of patients. Hypertension is a common complication of geriatric HF patients and might increase the post-operative death risk. However, our study found that hypertension was not a risk factor in post-operative mortality of geriatric HF patients, which might be attributed to the increasing awareness of the importance of blood pressure control in geriatric patients. Consistent with the literature (41), our studies also demonstrated that COPD and history of malignancy could increase the post-operative death risk of geriatric HF patients. Furthermore, Cha et al. (42) demonstrated that the post-operative mortality rate in severe-to-very severe COPD patients was significantly higher than those in the mild-to-moderate COPD patients. Therefore, a personalized and multi-disciplinary treatment strategy is recommended for geriatric HF patients.

**Table 5:** The risk factors of post-operative 1-year mortality in hip fracture patients in previous studies.

**Study (year)**	**Country**	**Number of patients**	**Fracture type**	**Post-operative 1-year mortality**	**Risk factors**
					
Dubljanin-Raspopović et al. (49)	Serbia	228	Hip fracture	25%	Lower motor Functional Independence Measure (FIM) score
Heyes et al. (35)	UK	465	Hip fracture	15.1%	Time to surgery ≥36 h
Bingol et al. (38)	Turkey	241	Hip fracture	25.3%	Neutrophil-to-lymphocyte ratio, monocyte-to-lymphocyte ratio
Fakler et al. (37)	Germany	209	Femoral neck fracture	23%	C-reactive protein
Folbert et al. (36)	The Netherlands	850	Hip fracture	23.2%	Male, age, higher ASA score, higher CCI, malnutrition, physical limitations in activities of daily living, decreasing Barthel Index
Mellner et al. (39)	Umeå	292	Femoral neck fracture	24%	Lower sernbo scores (based on age, habitat, mobility, and mental state)
Menéndez-Colino et al. (28)	Spain	509	Hip fracture	23.2%	Age, impairment in basic activities of daily living, cognitive impairment, malnutrition, anemia
Zanetti et al. (40)	Italy	1,211	Hip fracture	23.5%	Poor nutritional status (defined as MNA ≤ 23.5), increased cognitive, functional impairment
Gurger et al. (34)	Turkey	109	Hip fracture	22%	Delayed surgery, post-operative complications
Kim et al. (31)	South Korea	271	Hip fracture	23.4%	American society of anesthesiologists, time interval from trauma to operation
Hori et al. (32)	USA	428	Hip fracture	17.1%	Increased age, male sex, higher Charlson comorbidity index score, primary insurance status-Medicare/Medicaid, lower body mass index
Huette et al. (29)	France	309	Hip fracture	23.9%	Age, Lee score ≥3, time to surgery over 48 h
Canbeyli et al. (30)	Turkey	191	Intertrochanteric fracture	23.6%	Higher ASA grade, male sex, general anesthesia, and hemiarthroplasty procedures
Dobre et al. (33)	Romania	2,742	Hip fracture	29.72%	Age, male sex, length of stay in hospital, day of surgery, post-surgical complications, and late surgery

Additionally, our results demonstrated that hemoglobin and albumin levels were significantly associated with the 1-year post-operative mortality rate, which is supported by a previous study (43, 44). Delaying surgery in geriatric HF patients increased the risk of post-operative mortality, which has been noted in previous studies (45, 46). The time from injury to surgery consists of the time from injury to admission and the time from admission to surgery. Currently, early hip surgery in geriatric patients after admission has been widely accepted by clinicians. Thus, clinicians should pay attention to the early hip surgery and the time from injury to admission. In our opinion, improving the transfer efficiency of geriatric trauma patients could shorten the time from injury to admission and decrease the risk of post-operative mortality. Interestingly, our study found that the perioperative blood transfusion was also a risk factor of post-operative death in geriatric HF patients. There has been controversy about whether perioperative blood transfusion would increase post-operative mortality (47, 48). Transfusion in patients treated operatively for HF is associated with enhanced cardiovascular risk during the perioperative phase.

Some limitations of this study must be acknowledged. First, only geriatric HF patients who underwent surgery were examined, rather than all geriatric HF patients. Second, our study was retrospective. In our study, we included as many clinical variables as possible, but there were still a few variables that were not included, such as cardiovascular disease, C-reactive protein, thyroid-stimulating hormone, anesthesia technique, etc. Further studies are needed to investigate whether adding these clinical variables could improve the accuracy of the prediction model. Third, datasets in our study were unbalanced and not large enough. Further studies with large multicenter samples are needed to improve the accuracy of the random forest prediction model.

## Conclusion

Our study constructed a risk prediction model with high accuracy to predict the post-operative clinical prognosis of geriatric HF patients by the random forest algorithm. The random forest algorithm-based prediction model in our study could be used for the early identification of geriatric HF patients with a high risk of post-operative death and can provide important insights to doctors and nursing staff who can then adapt their diagnosis and treatment per patient by predicting risks in advance.

## Data Availability Statement

The original contributions presented in the study are included in the article/supplementary material, further inquiries can be directed to the corresponding author/s.

## Ethics Statement

This study was approved by the Institutional Ethical Review Board of West China Hospital, Sichuan University. The patients/participants provided their written informed consent to participate in this study.

## Author Contributions

FX wrote the manuscript. FX, RL, and ML collected the data and assisted in the data analysis. XD, ZZ, and ZX designed and supervised this project. All authors contributed to the article and approved the submitted version.

## Funding

This work was supported by the National Natural Science Foundation of China (31870961 and 81501879), the Sino-German Center for Research Promotion (GZ1219), the Science and Technology Department of Sichuan Province (Grant Nos. 2015HH0049, 2017SZ0127, and 2022YFS0099), and the National Clinical Research Center for Geriatrics, West China Hospital, Sichuan University (Z2018A11).

## Conflict of Interest

The authors declare that the research was conducted in the absence of any commercial or financial relationships that could be construed as a potential conflict of interest.

## Publisher's Note

All claims expressed in this article are solely those of the authors and do not necessarily represent those of their affiliated organizations, or those of the publisher, the editors and the reviewers. Any product that may be evaluated in this article, or claim that may be made by its manufacturer, is not guaranteed or endorsed by the publisher.
